# Structural network topology relates to tissue properties in multiple sclerosis

**DOI:** 10.1007/s00415-018-9130-2

**Published:** 2018-11-22

**Authors:** Svenja Kiljan, Kim A. Meijer, Martijn D. Steenwijk, Petra J. W. Pouwels, Menno M. Schoonheim, Geert J. Schenk, Jeroen J. G. Geurts, Linda Douw

**Affiliations:** 1 Department of Anatomy and Neurosciences, Amsterdam Neuroscience, MS Center Amsterdam, Amsterdam UMC, Location VU University Medical Center, De Boelelaan 1108, 1081 HZ Amsterdam, The Netherlands; 2 Department of Neurology, Amsterdam Neuroscience, MS Center Amsterdam, Amsterdam UMC, Location VU University Medical Center, Amsterdam, The Netherlands; 3 Department of Radiology and Nuclear Medicine, Amsterdam Neuroscience, MS Center Amsterdam, Amsterdam UMC, Location VU University Medical Center, Amsterdam, The Netherlands; 4 Department of Radiology, Athinoula A. Martinos Center for Biomedical Imaging, Massachusetts General Hospital/Harvard Medical School, Charlestown, MA USA

**Keywords:** Integration, Segregation, Histopathology, Post-mortem MRI, Axonal density, Neuronal size

## Abstract

**Objective:**

Abnormalities in segregative and integrative properties of brain networks have been observed in multiple sclerosis (MS) and are related to clinical functioning. This study aims to investigate the micro-scale correlates of macro-scale network measures of segregation and integration in MS.

**Methods:**

Eight MS patients underwent post-mortem in situ whole-brain diffusion tensor (DT) imaging and subsequent brain dissection. Macro-scale structural network topology was derived from DT data using graph theory. Clustering coefficient and mean white matter (WM) fiber length were measures of nodal segregation and integration. Thirty-three tissue blocks were collected from five cortical brain regions. Using immunohistochemistry micro-scale tissue properties were evaluated, including, neuronal size, neuronal density, axonal density and total cell density. Nodal network properties and tissue properties were correlated.

**Results:**

A negative correlation between clustering coefficient and WM fiber length was found. Higher clustering coefficient was associated with smaller neuronal size and lower axonal density, and vice versa for fiber length. Higher whole-brain WM lesion load was associated with higher whole-brain clustering, shorter whole-brain fiber length, lower neuronal size and axonal density.

**Conclusion:**

Structural network properties on MRI associate with neuronal size and axonal density, suggesting that macro-scale network measures may grasp cortical neuroaxonal degeneration in MS.

**Electronic supplementary material:**

The online version of this article (10.1007/s00415-018-9130-2) contains supplementary material, which is available to authorized users.

## Introduction

Multiple sclerosis (MS) is a disease of the central nervous system characterized by demyelination in white matter (WM) and gray matter (GM), accompanied by neurodegeneration. Clinically, MS patients show both motor dysfunction and cognitive impairment. Several attempts have been made to understand how these symptoms arise from radiologically observed brain damage. Disconnection of brain regions due to WM and GM damage disturbs the optimal information flow through the brain and hence has been proposed to be a substrate of clinical disability in MS [7, 16, 17]. Innovative computational methods originating from graph theory have enabled researchers to discover topological patterns of brain connectivity that contribute to optimal information distribution through the brain in healthy subjects [1, 2, 28, 29]. Moreover, in disease, this approach has been used to explain clinical symptoms and model disease progression [1].

In MS, impaired structural network organization, especially of segregative and integrative properties, has been observed. Changes in these macroscopic structural network properties relate to WM lesion load and clinical disability [27], occur before functional network alterations are present [26], and precede clinical impairment in early MS [10]. Furthermore, using machine learning, clinically isolated syndrome and relapsing remitting MS patients could be distinguished based on their structural brain network properties [18]. Despite the clinical relevance of structural network changes, their histological correlates in MS remain elusive. Recently it has been shown that microscopic tissue characteristics (*e.g*. spine density) are correlates of macroscopic network properties (*e.g*. clustering coefficient) in humans, monkeys and mice: separate datasets with information on cellular and network properties of different GM regions were collated to investigate their relationship [23, 24, 34, 35]. Studying the cellular correlates of network changes within patients could be a first step towards a better understanding of network alterations in MS.

Therefore, we investigated the microscopic histological correlates of macroscopic network measures of segregation and integration in MS. To do so, we obtained a post-mortem dataset consisting of in situ brain MRI and histological brain tissue characteristics from the same subjects. We first described the relationship between nodal measures of macro-scale segregation and integration (i.e. clustering coefficient and WM fiber length). Second, we identified microscopic cellular correlates (i.e. neuronal size, neuronal density, axonal density and total cell density) of these macroscopic network measures. Finally, we examined the associations between micro-scale tissue characteristics and the macro-scale measures of segregation and integration, and the effect of WM lesion load on these measures.

## Methods

### Subjects

Eight MS patients were included (Table 1). Of each patient, histological data and in situ post-mortem (PM) MRI data were collected with a short PM delay (for more information see Online Resource 1). This data was collected in collaboration with the Netherlands Brain Bank. The study was approved by the institutional ethics review board. Before death, the MS patients or their next of kin provided written informed consent for the use of their tissue and clinical information for research purposes to the Netherlands Brain Bank. Furthermore, an in vivo imaging dataset of eight age- and sex-matched healthy subjects was included to construct a structural connectivity atlas. This group consisted of 5 males and 3 females with a median age of 61.5 years [range 59–63], age and sex did not statistically differ between groups. Written informed consent was also obtained from all healthy subjects.

**Table 1:** Demographics of included patients with multiple sclerosis

Case	Sex	Age (y)	Post-mortem delay (h)	Cause of death
1	M	51	4.0	Pneumonia
2	F	57	3.5	Euthanasia
3	M	56	5.0	Gastric perforation
4	M	53	4.0	Euthanasia
5	F	56	4.0	Pneumonia
6	F	81	2.5	Cachexia
7	M	80	4.5	Pneumonia
8	M	71	4.0	Pneumonia
	5:3 [M:F]	56.5 [51–81]	4.0 [2.5–5]	

Median [min–max] is provided for age and post-mortem delay in the bottom row

*M* male, *F* female, *y* years, *h* hours

### Post-mortem MRI acquisition

For each MS patient, post-mortem in situ whole-brain MRI was acquired using a 1.5T with an 8-channel head coil (for more information see Online Resource 1). The protocol included a dual-echo T2-weighted sequence to determine WM lesion volumes, a 3DT1-weighted fast spoiled gradient echo (FSPGR) sequence and a 2D echo-planar diffusion tensor imaging (DTI) (for more information see Online Resource 1).

### Construction of a group-based structural connectivity atlas in healthy subjects

**Fig. 1:**
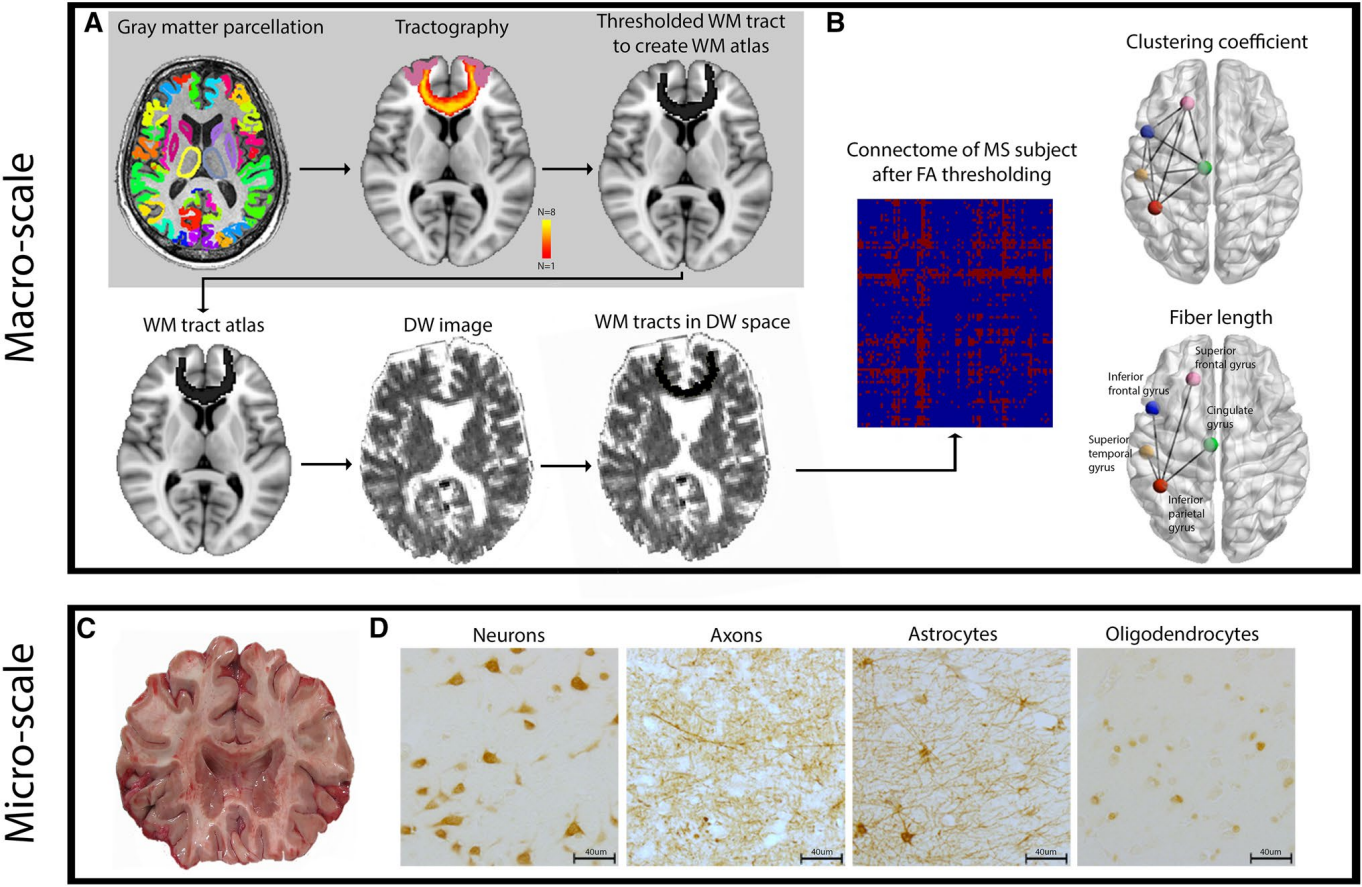
Overview of the study workflow. **a** Displays the methodological pipeline to compute connectomes in investigated subjects. The gray background surrounding the upper panels indicates the pipeline in healthy subjects. Upper left picture displays the 92 parcellated gray matter regions that were used to build the connectome. The upper middle picture shows an example of a tract between the left and right superior frontal gyrus. The color scale indicates the presence of voxels in this tract across the eight healthy subjects. Yellow indicates presence of the tract in a voxel in eight out of eight subjects while red indicates presence of a tract in a voxel in one out of eight subjects. The right upper picture shows the thresholded and binarized white matter tract, only containing voxels that were present in six out of eight subjects. This step was performed for all 92 × 91 white matter connections resulting in a white matter tract atlas, displayed in a simplified manner in the lower left panel. The white matter tract atlas was registered to subject space and then co-registered to the native diffusion-weighted (DW) image, as shown in the lower middle and left panels. **b** Clustering coefficient and fiber length are schematically displayed in a glass brain in the upper and lower left panels, respectively. Fiber length is depicted here as the Euclidian distance for illustration purposes, note that it was measured as the actual fiber length. The left panel of B shows the 20% density thresholded connectivity matrix of 1 subject with multiple sclerosis based on the mean FA underneath the white matter connections. **c** Shows an example of a 1 cm thick coronal brain slice from which brain regions of interest (ROIs) tissue blocks were dissected. ROIs are indicated in the lower panel glass brain in **b. d** The histological staining of neurons, axons, astrocytes and oligodendrocytes. The sum of the number of astrocytes, oligodendrocytes and neurons was used as the total cell density

A structural connectivity atlas was constructed in healthy subjects to overcome the potentially confounding effect of MS related WM lesions on tractography methods. A 3T MRI system (General Electrics, USA) was used to acquire 3DT1 and diffusion weighted images (for more information see Online Resource 1). Diffusion-weighted images were corrected for motion and eddy current distortion using FMRIB’s Diffusion Toolbox (FSL-FDT; part of FSL 5.0.9 https://fsl.fmrib.ox.ac.uk/fsl/fslwiki [14]). Cortical GM was segmented using the automated anatomical labeling (AAL) atlas [33] and FIRST (part of FSL) was used to delineate deep GM, resulting in a total of 92 nodes. First, bedpostx was run to build up diffusion parameter distributions at each voxel, after which probabilistic tractography was conducted (probtrackx2, part of FSL, 5000 streamlines per voxel) to obtain probabilistic maps of WM connections running between all pairs of nodes resulting in a structural network for each subject (Fig. 1a; for more information see Online Resource 1).

### Construction of individual structural connectomes in MS patients

The post-mortem in situ diffusion images were corrected for motion and eddy current distortion using FSL-FDT. Then the diffusion tensor was fitted and fractional anisotropy (FA) was computed for each voxel. Structural networks of individual MS patients were constructed by applying the healthy subject based structural connectivity atlas to their FA maps. To optimize registration pipelines, all WM lesions were manually outlined on the T2 images and lesion filling of 3DT1-weighted images was performed (for more information see Online Resource 1). Subsequently, the structural connectivity atlas was non-linearly co-registered to lesion filled 3DT1-weighted images and then linearly registered to diffusion-weighted images for every patient [5, 6, 30]. To ensure the inclusion of WM only, masks of each WM connection was multiplied with each patients’ lesion-filled WM mask derived from SIENAX. A connectivity matrix with mean FA values per connection was computed for every MS patient. To obtain a binary structural connectivity matrix per patient, the 20 percent [8] WM connections with the highest mean FA values were selected for each individual (Fig. 1B).

### Computation of macro-scale network topology

Using the structural connectivity matrix of each subject, macro-scale network topology was computed for each of the 92 regions using the Brain Connectivity Toolbox [22] and in-house developed Matlab scripts (Matlab version 13a, Mathworks, Natick, MA, USA). To measure segregation, we computed the clustering coefficient (Fig. 1b, upper panel). For integration, we used the average fiber length of all WM connections of a node (Fig. 1b, lower panel; for more information see Online Resource 1). The average WM fiber length per node was computed for every MS subject based on their individual connectivity matrix (for more information see Online Resource 1). Whole-brain measures of the clustering coefficient and fiber length were calculated by averaging the values of all nodes for each patient.

### Tissue selection and cellular micro-scale measures

To quantify micro-scale regional features, five cortical brain regions were excised according to a standardized protocol [25]. The superior frontal gyrus (*N* = 8), inferior frontal gyrus (*N* = 6), cingulate gyrus (*N* = 7), inferior parietal lobule (*N* = 7) and superior temporal gyrus (*N* = 7) were excised, adding up to a total of 33 tissue blocks across all patients (Fig. 1c; for more information see Online Resource 1). From now on these will be referred to as regions of interest (ROIs). Tissue blocks were formalin fixed and embedded in paraffin and tissue sections were cut at 10 µm. Four different micro-scale measures were assessed: (1) neuronal density (i.e. neuronal count per mm^2^), (2) neuronal size in µm^2^ (i.e. total area stained for neurons divided by the neuronal number, per mm^2^), (3) axonal density in relative optical density (ROD) and (4) total cell density (i.e. the sum of the number of astrocytes, neurons and oligodendrocytes in number per mm^2^; Fig. 1d). Myelin density was obtained to identify whether it was related to the above mentioned micro-scale measures.

### Staining procedure

Immunohistochemistry was performed to stain for neurons, axons, astrocytes and myelin (for more information see Online Resource 1). Antigen retrieval was performed prior to staining using a citrate buffer (pH 6) for all stainings, except for the olig2 staining where Tris–EDTA (pH 9) was used as a pre-treatment. Sections were blocked with normal goat serum and after incubation with the primary antibodies sections were rinsed and incubated with biotin labeled secondary antibodies (1:500 DAKO, Glostrup, Denmark) then they were rinsed again and incubated with streptavidin-biotin-peroxidase complexes (1:200; Vectastain; Vector Laboratories Inc., Burlingame, CA, USA). Only during the olig2 staining Envision horseradish peroxidase complexes (DAKO, Glostrup, Denmark) was used instead of a regular biotinylated secondary antibodies and streptavidin-biotin-peroxidase complexes. Finally, sections were rinsed and 3,3′-diaminobenzidine tetrahydrochloride dihydrate (DAB; DAKO, Glostrup, Denmark) precipitate was generated in reaction with peroxidase.

### Acquisition and quantification of cellular micro-scale measures

Images of were acquired using a Leica DM/RBE photomicroscope (Leica, Heidelberg, Germany). A 4 × 4 mm^2^ grid was overlaid on an entirely imaged Nissl stained section and by means of random systematic sampling grid frames containing six layered cortex were selected as quantification sites for every tissue block. These sites were the same for all micro-scale measures quantified in consecutive sections per tissue block. Neuronal density, size and astrocyte density were quantified using MCID segmentation scripts (MCID Image Analysis Software Solutions for Life Sciences, UK), while the number of oligodendrocytes was quantified using ImageJ software. Axonal density was quantified using the relative optical density (ROD). ROD is a method that measures the amount of stained structures based on the optical staining density by converting an image to grayscale and correcting for the background intensity [19]. Finally, the extent of cortical (de)myelination was also quantified using the ROD.

### Statistical analysis

Statistical analyses were performed using Matlab and SPSS (version 22.0, IBM, Chicago, IL, USA). Spearman’s rank correlation coefficient was used to evaluate the correlations between (a) macro-scale network properties, (b) regional macro-scale network properties with their corresponding micro-scale histological features and (c) whole brain WM lesion volume and regional GM demyelination with macroscopic and microscopic measures. In the above-described correlation data points were interdependent since different data points (i.e. different brain regions) were used from the same subjects. Therefore, we also performed this analysis with averaged macroscopic and microscopic characteristics of the every ROI per patient. To evaluate the possible effect of age, we performed a partial correlation including age as a covariate. *P* values were considered significant at *p* < 0.050 and two-tailed testing was performed. In addition to these non-parametric statistics, we performed permutation testing (for more information see Online Resource 1) to further objectify the significance of the correlations between micro-scale and macro-scale measures. Furthermore, with respect to the connection density threshold for each individual connectome (20% in our main analyses), reproducibility of all results was tested across different density thresholds (namely 20%, 25% and 30%). Finally, we quantified whether the observed correlations were region specific or merely a global property of the network, and therefore, true for all regions through random resampling (for more information see Online Resource 1).

## Results

### Macro-scale features of segregation and integration in MS

**Fig. 2:**
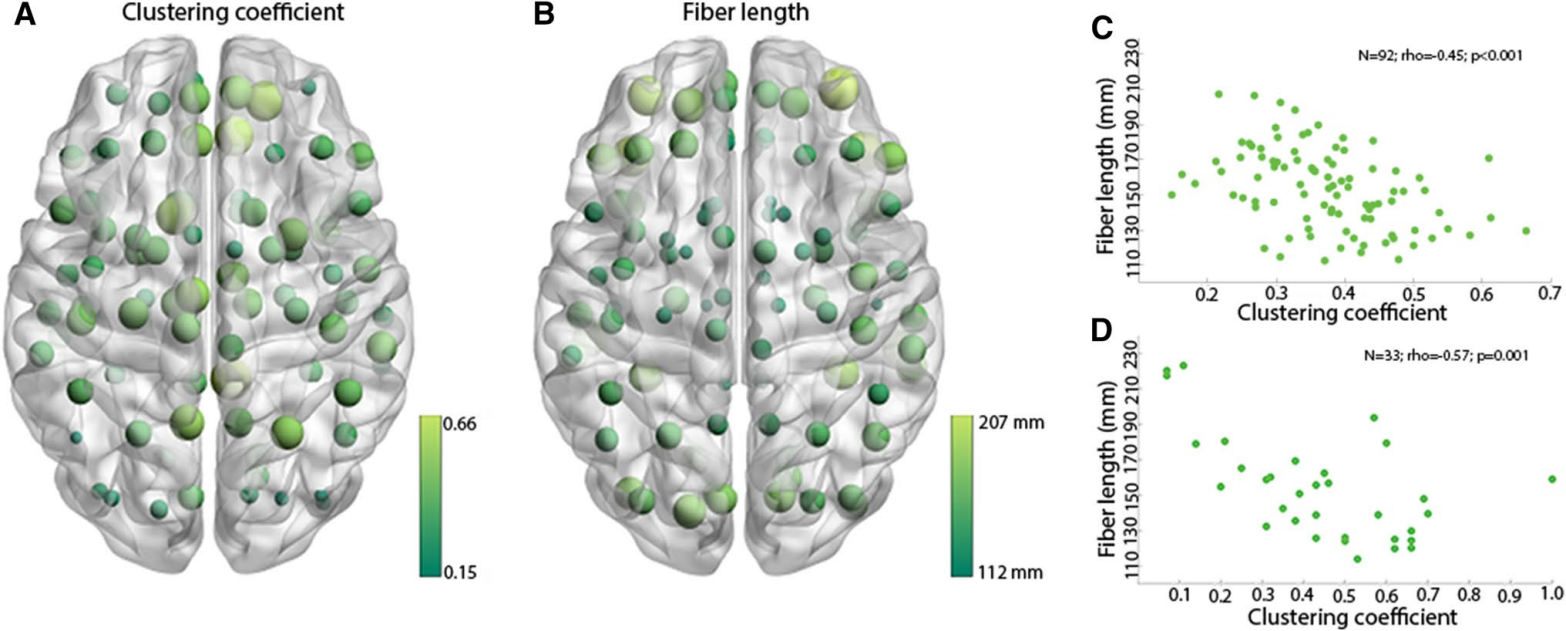
Anti-correlation between macroscopic clustering coefficient and fiber length. **a** Shows the mean clustering coefficient of all 92 parcellated gray matter regions across the eight subjects. Light green and large dots represent high clustering coefficients. Dark green and small dots represent low clustering coefficients. **b** Shows the mean fiber length of all 92 parcellated gray matter regions across the eight subjects. Light green and large dots represent long fiber lengths. Dark green and small dots represent short fiber lengths. **c** Shows the anti-correlation between macroscopic clustering coefficient and fiber length displayed in **a, b. d** Anti-correlation between macroscopic clustering coefficient and fiber length of the 33 ROIs

Clustering coefficient was negatively correlated with fiber length across all 92 nodes of the connectome (*N* = 92; rho = − 0.45; *p* < 0.001; Fig. 2a–c). To assess whether the ROIs had similar properties on the macro-scale compared to all other nodes in the connectome, we performed the same correlation for this subset of regions. Again, a negative association was present within ROIs when correlating their clustering coefficient and fiber length (*N* = 33; rho = − 0.57; *p* = 0.001; Fig. 2d).

### Macro-scale network characteristics are reflected by cellular micro-scale features in MS patients

**Fig. 3:**
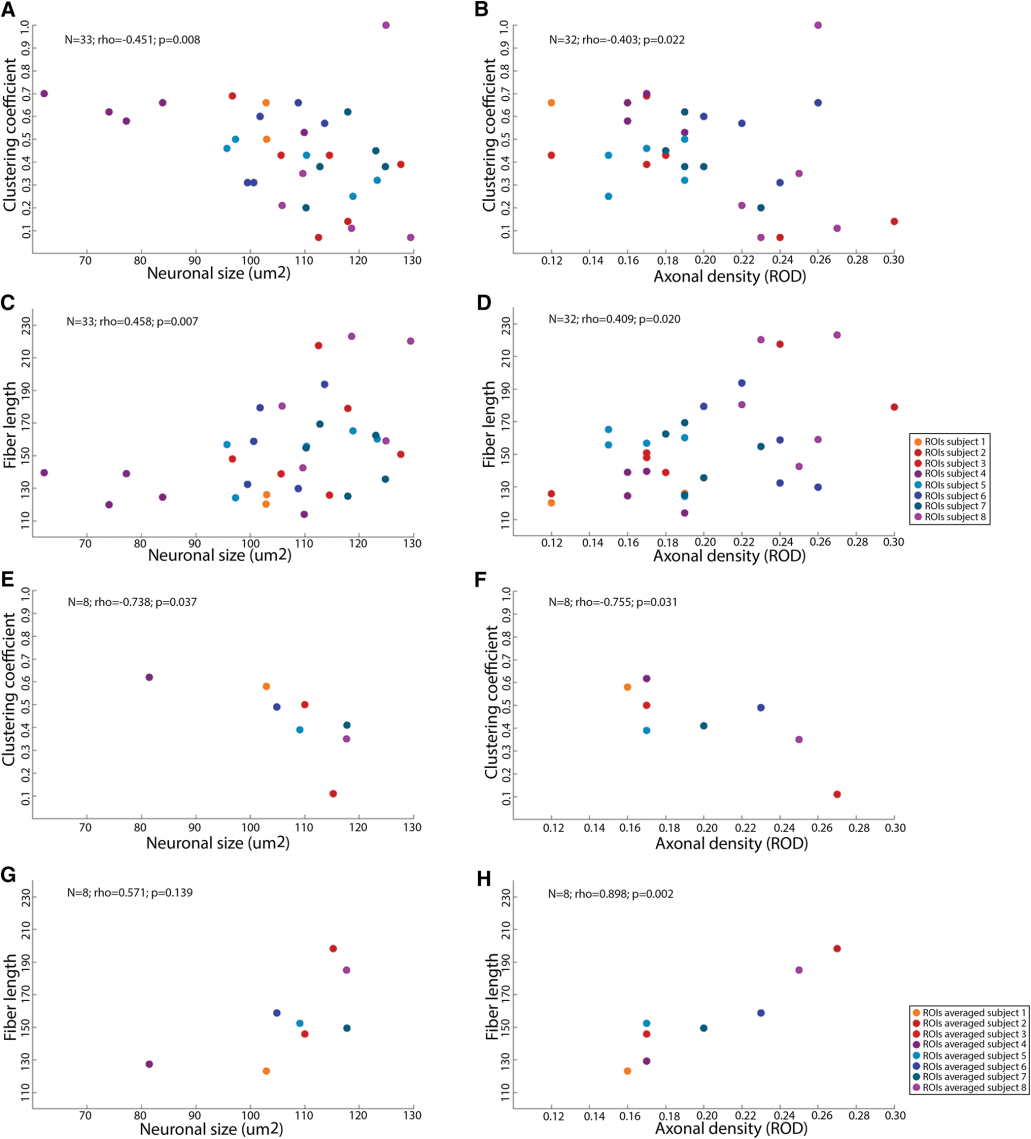
Macroscopic clustering coefficient and fiber length correlate with microscopic cellular features. **a** Correlation between clustering coefficient and neuronal size. **b** Correlation between clustering coefficient and axonal density. **c** Correlation between fiber length and neuronal size. **d** Correlation between fiber length and axonal density. **e** Correlation between clustering coefficient and neuronal size. Their values of different ROIs are averaged for every person. **f** Correlation between clustering coefficient and axonal density. Their values of different ROIs are averaged for every person. **g** Correlation between fiber length and neuronal size. Their values of the ROIs are averaged for every person. **h** Correlation between fiber length and axonal density. Their values of different ROIs are averaged for every person. The scatterplot dots are ROIs and their color indicates to which subject they belong in **a**–**d**. In **e**–**h** the macro-scale and micro-scale information on ROIs was averaged and the color of the dots indicate to which subject to averaged ROIs belong

We found significant correlations between both clustering coefficient and fiber length with neuronal size and axonal density were found (Fig. 3a–d). In particular, higher macro-scale regional clustering coefficient was correlated with smaller neuronal size (*N* = 33; rho = − 0.451; *p* = 0.008; Fig. 3a) and lower micro-scale axonal density (*N* = 32; rho = − 0.403; *p* = 0.022; Fig. 3b), while longer average fiber length was correlated with a larger neuronal size (*N* = 33; rho = 0.458; *p* = 0.007; Fig. 3c) and higher axonal density (*N* = 32; rho = 0.409; *p* = 0.020; Fig. 3d). These results remained significant after permutation testing. In addition, similar results were obtained after replication with density levels of 25% and 30% (data not shown). No significant effect of age was detected regarding correlations between clustering coefficient and neuronal size and between fiber length and axon density (*N* = 33; rho = − 0.357; *p* = 0.048 and *N* = 32; rho = 0.402; *p* = 0.025, respectively). After correction for age, correlations between clustering coefficient and axon density and between fiber length and neuronal size were no longer statistically significant (*N* = 32; rho = 0.316; *p* = 0.084 and *N* = 33; rho = 0.311; *p* = 0.089, respectively).

The analysis was repeated with averaged macroscopic and microscopic characteristics of the every ROI per patient (Fig. 3e–h). A significant correlation was still found between clustering coefficient and neuronal size (*N* = 8; rho = − 0.738; *p* = 0.037; Fig. 3e), clustering coefficient and axonal density (*N* = 8; rho = − 0.755; *p* = 0.031; Fig. 3f) and fiber length and axonal density (*N* = 8; rho = 0.898; *p* = 0.002; Fig. 3h). The correlation between fiber length and neuronal size was not significant (*N* = 8; rho = 0.571; *p* = 0.139; Fig. 3g). Table 2 displays network characteristics per patient: an overview of the mean and range of macro-scale network measures for all 92 GM regions and for the ROIs per subject. It also includes the mean range of micro-scale measures of ROIs and anatomical regions that were dissected per patient. Note that macro-scale network characteristics of ROIs cover a small range of possible within subject macro-scale measures, although across subjects a large range of macroscopic measures is covered. In addition, the range of micro-scale measures covered by the different ROIs within every subject is small, ensuring the validity of our results when averaging data points within subjects.

**Table 2:** Macroscopic, microscopic and lesion load measures per patient

Patient ID	Range of macroscopic and microscopic measures within the ROIs				Range of macroscopic measures of the 92 Gy matter regions		Whole brain T2 lesion load (mL)	Brain regions included as ROIs
	Fiber length (mm)	Clustering coefficient	Axonal density (ROD)	Neuronal size (um^2^)	Fiber length (mm)	Clustering coefficient		
1	123.17 [120.26–126.07]	0.58 [0.50–0.66]	0.16 [0.12–0.19]	102.95 [102.90–103.00]	134.75 [85.80–231.24]	0.51 [0.00–1.00]	28.81	CG, SFG
2	198.23 [178.95–217.51]	0.11 [0.07–0.14]	0.27 [0.24–0.30]	115.54 [112.55–117.92]	172.16 [112.18–250.10]	0.09 [0.00–0.52]	10.84	SFG, STG
3	143.38 [125.78–150.82]	0.43 [0.39–0.69]	0.17 [0.12–0.18]	110.11 [96.71–127.65]	155.74 [111.13–238.73]	0.41 [0.00–1.00]	17.43	CG, IFG, SFG, STG
4	124.48 [114.00–139.55]	0.62 [0.53–0.70]	0.17 [0.16–0.19]	77.22 [62.13–109.91]	127.19 [81.43–222.68]	0.53 [0.00–1.00]	32.10	CG, IFG, SFG, STG, IPL
5	156.78 [124.14–165.22]	0.43 [0.25–0.50]	0.17 [0.15–0.19]	110.33 [95.71–123.32]	150.49 [92.91–218.917]	0.33 [0.00–1.00]	25.45	CG, IFG, SFG, STG, IPL
6	158.78 [129.77–193.72]	0.57 [0.31–0.66]	0.24 [0.20–0.26]	101.79 [99.49–113.65]	149.86 [98.41–213.48] 171.50	0.47 [0.00–1.00]	16.16	CG, IFG, SFG, STG, IPL
7	145.16 [125.13–169.32]	0.38 [0.20–0.62]	0.20 [0.18–0.23]	115.35 [110.24–124.81]	153.89 [55.48–225.45] 171.50	0.33 [0.00–0.80]	5.99	CG, IFG, SFG, STG, IPL
8	180.42 [142.50–223.16]	0.21 [0.07–1.00]	0.25 [0.22–0.27]	118.60 [105.85–129.45]	165.48 [110.22–252.54]	0.20 [0.00–1.00]	5.97	CG, IFG, SFG, STG, IPL

The median is shown followed by ranges between brackets of fiber length, clustering coefficient, axonal density and neuronal size are presented for the ROIs, per person; the median and ranges are also shown for fiber length and clustering coefficient for the 92 Gy matter regions

*ROIs* regions of interest, *ROD* relative optical density, *mL* milliliter, *mm* millimeter, *CG* cingulate gyrus, *SFG* superior frontal gyrus, *STG* superior temporal gyrus, *IFG* inferior frontal gyrus, *IPL* inferior parietal gyrus

**Fig. 4:**
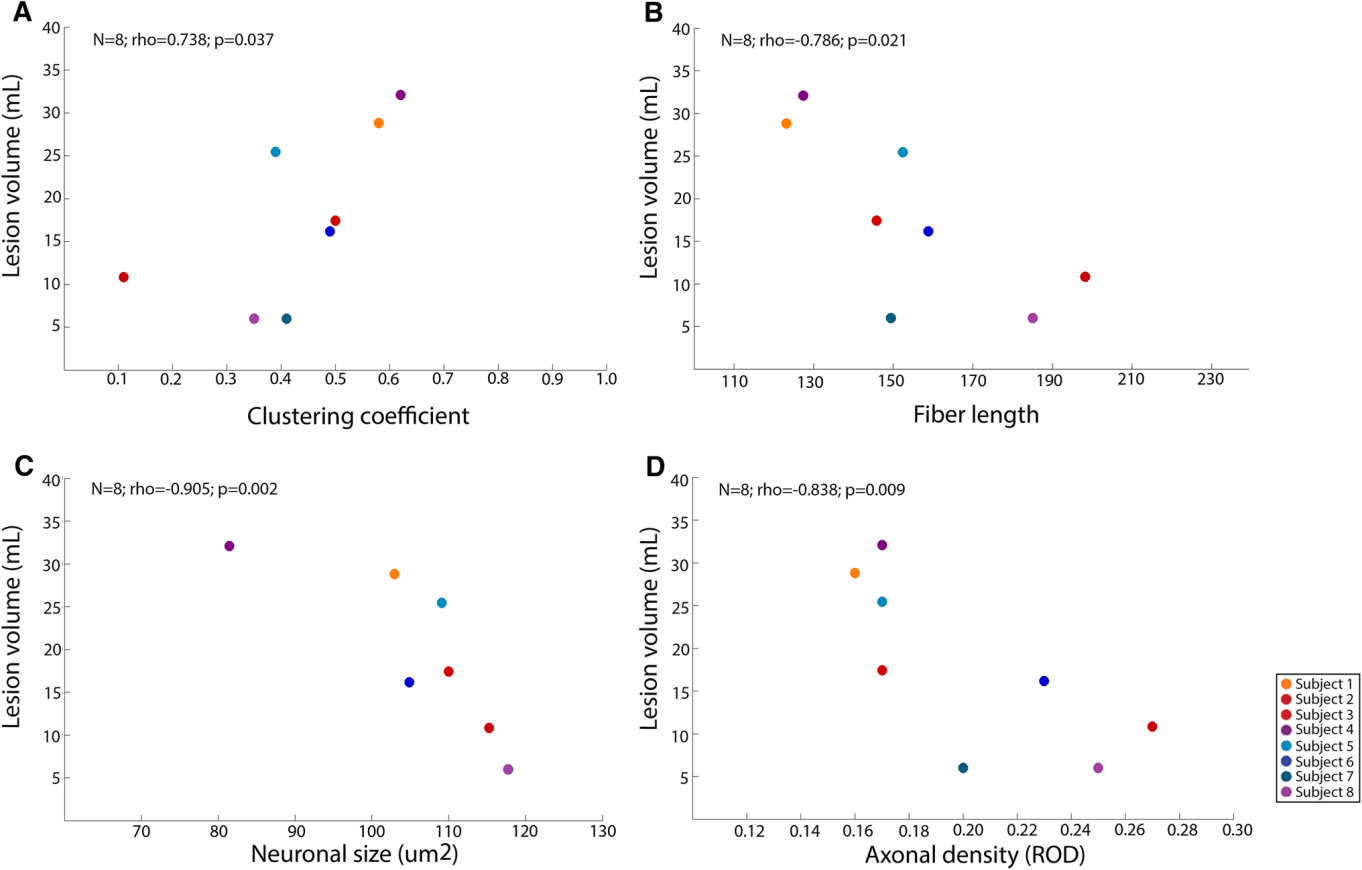
WM lesion volume correlated with macroscopic network properties and microscopic tissue characteristics. **a** Correlation between clustering coefficient and white matter lesion volume. Whole-brain measures were used for both measures for every person. **b** Correlation between fiber length and white matter lesion volume. Whole-brain measures were used for both measures for every person. **c** Correlation neuronal size and white matter lesion volume. Whole-brain lesion volume was used for every person while micro-scale measures were averaged across regions of interest (ROIs). **d** Correlation between axonal density and white matter lesion volume. Whole-brain lesion volume was used for every person while micro-scale measures were averaged across regions of interest (ROIs). The scatterplot dot colors indicate the different subjects

No associations were found between nodal GM demyelination and macro-scale measures of integration and segregation or micro-scale measures of axonal density and neuronal size. On the contrary, whole-brain WM lesion volume was associated with clustering coefficient, fiber length, neuronal size and axonal density (*N* = 8; rho = 0.738; *p* = 0.037; rho = − 0.786; *p* = 0.021; *N* = 8; rho = − 0.905; *p* = 0.002; *N* = 8; rho = − 0.838; *p* = 0.009; *N* = 8, respectively; Fig. 4a–d).

### Macro-scale connectomic measures and micro-scale cellular features are node specific

After resampling of the 92 macro-scale network indices within each patient, such that these were randomly coupled to the patient’s nodal micro-scale characteristics (*N* = 2–5), over all 1000 repetitions, the association between macro-scale clustering coefficient and micro-scale neuronal size had a lower correlation coefficient and a higher *p* value (i.e. did not reach significance) (rho = − 0.299; *p* = 0.188) compared to the actual micro-scale and macro-scale correlation (*N* = 33; rho = − 0.451; *p* = 0.008). This indicates that the micro-scale properties of a ROI did not solely represent global network features of a subject, but that they were specific to the macro-scale properties of that ROI.

## Discussion

This study provides novel information regarding the relationship of segregative and integrative regional macro-scale network properties and their micro-scale histological correlates in MS. We showed that a negative correlation exists between nodal features of segregation (i.e. clustering coefficient) and integration (i.e. fiber length) at the macro-scale. Further, these macro-scale network properties were associated with neuronal size and axonal density. Cortical regions with higher clustering coefficients were characterized by smaller neurons and lower axonal density, while regions with a longer fiber length contained larger neurons and higher axonal densities. Finally, subjects with a higher WM lesion volumes showed higher whole-brain clustering and shorter whole-brain fiber length but also smaller neurons and a lower axonal densities.

We found a negative correlation between segregation and integration on the macro-scale across all 92 nodes on a group-level. This indicates that regions with a high clustering coefficient are mostly connected to other regions via relatively short fibers, while regions that have long distance WM fibers have less connections between their nearest neighbors. From a graph theoretical point of view, a network with a trade-off between segregative and integrative properties may be optimal for information flow [20, 21]. Although alterations in the structural brain in MS have been described, maintained small world properties have been reported [12, 27]. Therefore, the negative association between clustering coefficient and fiber length in our dataset is in line with previous literature.

**Fig. 5:**
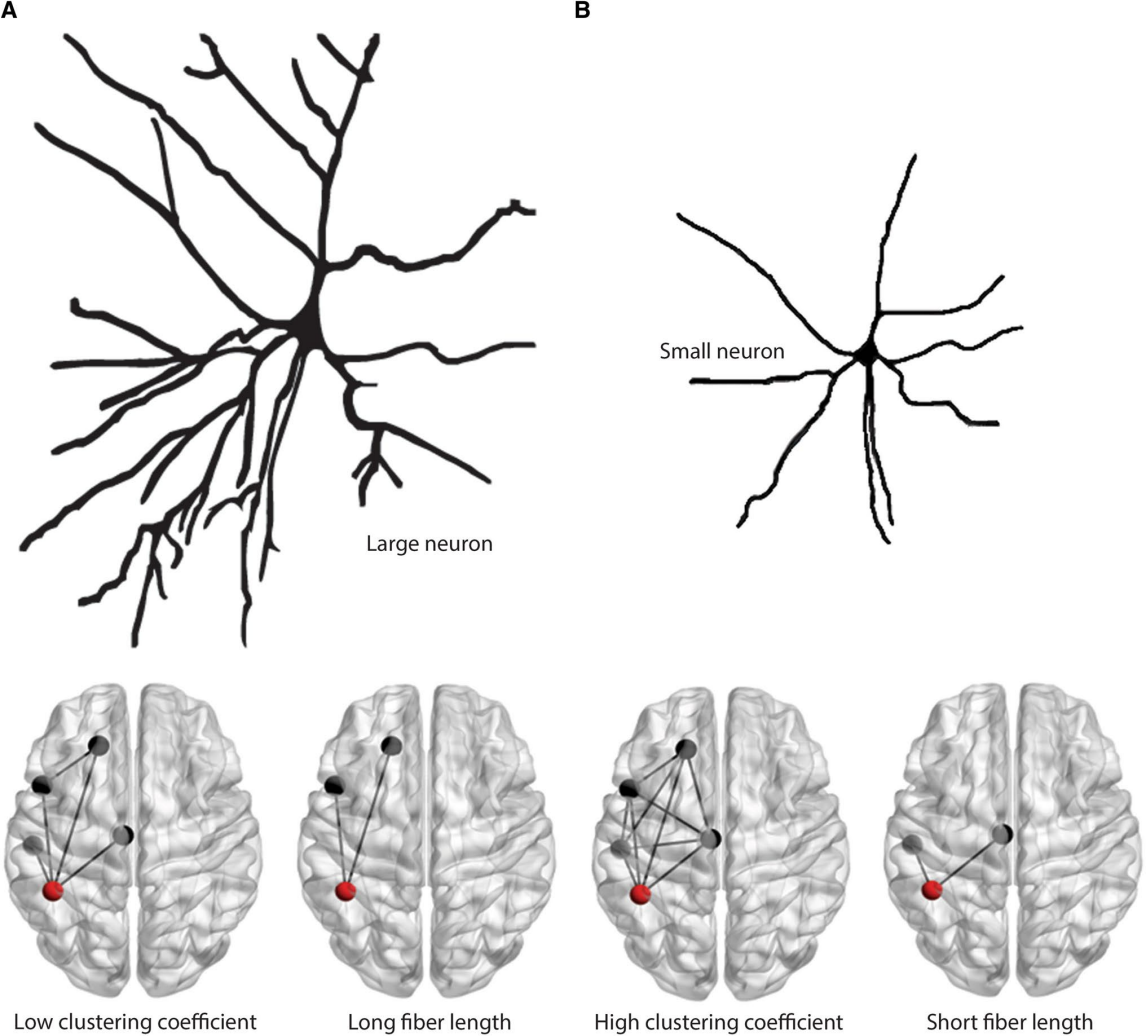
Macro-scale network measures are reflected by micro-scale features of neuronal morphology. **a** This panel displays our hypothesis that neurons with large cell bodies and extensive dendritic branching are present in regions that are macroscopically strongly involved in integrative processes (long fiber lengths) instead of local segregation (low clustering coefficients). **b** This panel displays our thesis that neurons with small cell bodies and limited dendritic branching are present in regions that are macroscopically strongly involved in segregation (high clustering coefficients) and less involved in global integration (short fiber lengths)

The next step was to investigate the micro-scale correlates of these macro-scale measures. Higher clustering coefficient was associated with smaller neuronal size and lower axonal density, and vice versa for fiber length. Studies have shown that micro-scale axonal density reflects projections between neurons of different cortical regions as well as recurrent connectivity between neurons within regions [31, 32]. Furthermore, neuronal size has been shown to reflect the extent of dendritic branching of a neuron [13]. Electrophysiological studies indicated that quantitative morphological differences of neurons (i.e. axonal and dendritic arborization) may contribute to their qualitative abilities of complex signal integration, the formation of integrative neural networks and associated cognitive processing [4, 15, 36]. This may be in line with our findings that neuronal size and axonal density are higher in regions that are macroscopically more involved in integrative rather than segregative processes and vice versa (Fig. 5).

Macro-scale and micro-scale measures were associated with whole brain WM lesion volume. This indicates that differences in these measures between patients are not solely a matter of inter-subject variation, but are at least partly explained by disease severity. More specifically, we found that higher WM lesion volumes were associated with higher whole-brain clustering coefficient and lower whole-brain fiber length. This is in line with in vivo studies where structural network efficiency was associated with WM lesion volume [12, 27]. Zooming in, we found that a higher WM lesion volume, but not GM demyelination, was associated with lower neuronal size and lower axonal density. Interestingly, the latter two micro-scale measures are thought to represent the histopathological underpinnings of cortical atrophy in MS [19], which is also related to damage in WM tracts [3, 11, 30]. Additionally, future research should determine whether specifically smaller neurons tend to shrink since previous research indicates that they may preferentially atrophy due to higher susceptibility of their thin axons to degenerate after WM damage [9]. According to the literature it could be that WM lesions mediate both macro-scale network characteristics and micro-scale GM characteristics, however, we cannot support this finding based on our results. Finally, our findings indicate that measures of structural network organization may be seen as a bridge between micro-scale cortical alterations and clinical disability.

Some limitations were present in our study. First, we did not include healthy subjects in this study. In future studies, the inclusion of healthy subjects is necessary to evaluate whether the relation found between macroscopic and microscopic properties in this study are also present in healthy subjects. Furthermore, although our sample size is not small for a combined post-mortem and MRI study, future studies with larger samples are necessary to replicate our findings. Also, MS patients were imaged on two different MRI scanners, but since network measures are quantified from binarized WM tract FA values within every subject, scanner influence on network measures is minimal. Lastly, crossing fibers may be a confounding factor when determining 20% of tracts with the highest FA values. However, since the effect of crossing fibers would be present in all subjects, inter-subject variation and disease severity remain more important factors determining our results.

To conclude, we showed that in MS, a trade-off (i.e. negative correlation) exists between segregative and integrative properties of brain regions. Subjects with distinct macro-scale network characteristics also showed distinct tissue characteristics on the micro-scale. More specifically, regions containing smaller neurons and lower axonal densities were characterized by more locally clustered structural connectivity, while the opposite relation was observed for regions with longer WM connections. WM lesion volume was associated with network properties and tissue properties in MS. Our study can be seen as a first step to better understand what network characteristics, and possibly changes therein, commonly observed in MS may mean on a cellular level in MS.

## Electronic supplementary material

Below is the link to the electronic supplementary material.

Supplementary material 1 (DOCX 72 KB)

## References

[1] Bassett DS, Bullmore ET (2009). Human brain networks in health and disease. Curr Opin Neurol.

[2] Bassett DS, Sporns O (2017). Network neuroscience. Nat Neurosci.

[3] Bodini B, Chard D, Altmann DR, Tozer D, Miller DH, Thompson AJ, Wheeler-Kingshott C, Ciccarelli O (2016). White and gray matter damage in primary progressive MS: The chicken or the egg?. Neurology.

[4] Chklovskii DB (2004). Synaptic connectivity and neuronal morphology: two sides of the same coin. Neuron.

[5] Daams M, Steenwijk MD, Schoonheim MM, Wattjes MP, Balk LJ, Tewarie PK, Killestein J, Uitdehaag BM, Geurts JJ, Barkhof F (2016). Multi-parametric structural magnetic resonance imaging in relation to cognitive dysfunction in long-standing multiple sclerosis. Mult Scler.

[6] Daams M, Steenwijk MD, Wattjes MP, Geurts JJ, Uitdehaag BM, Tewarie PK, Balk LJ, Pouwels PJ, Killestein J, Barkhof F (2015). Unraveling the neuroimaging predictors for motor dysfunction in long-standing multiple sclerosis. Neurology.

[7] Dineen RA, Vilisaar J, Hlinka J, Bradshaw CM, Morgan PS, Constantinescu CS, Auer DP (2009). Disconnection as a mechanism for cognitive dysfunction in multiple sclerosis. Brain.

[8] Eavani H, Satterthwaite TD, Filipovych R, Gur RE, Gur RC, Davatzikos C (2015). Identifying Sparse Connectivity Patterns in the brain using resting-state fMRI. Neuroimage.

[9] Evangelou N, Konz D, Esiri MM, Smith S, Palace J, Matthews PM (2001). Size-selective neuronal changes in the anterior optic pathways suggest a differential susceptibility to injury in multiple sclerosis. Brain.

[10] Fleischer V, Groger A, Koirala N, Droby A, Muthuraman M, Kolber P, Reuter E, Meuth SG, Zipp F, Groppa S (2017). Increased structural white and grey matter network connectivity compensates for functional decline in early multiple sclerosis. Mult Scler.

[11] Haider L, Zrzavy T, Hametner S, Hoftberger R, Bagnato F, Grabner G, Trattnig S, Pfeifenbring S, Bruck W, Lassmann H (2016). The topography of demyelination and neurodegeneration in the multiple sclerosis brain. Brain.

[12] He Y, Dagher A, Chen Z, Charil A, Zijdenbos A, Worsley K, Evans A (2009). Impaired small-world efficiency in structural cortical networks in multiple sclerosis associated with white matter lesion load. Brain.

[13] Jacobs B, Schall M, Prather M, Kapler E, Driscoll L, Baca S, Jacobs J, Ford K, Wainwright M, Treml M (2001). Regional dendritic and spine variation in human cerebral cortex: a quantitative golgi study. Cereb Cortex.

[14] Jenkinson M, Beckmann CF, Behrens TE, Woolrich MW, Smith SM (2012). Fsl. Neuroimage.

[15] Knudsen EI (1994). Supervised learning in the brain. J Neurosci.

[16] Louapre C, Perlbarg V, Garcia-Lorenzo D, Urbanski M, Benali H, Assouad R, Galanaud D, Freeman L, Bodini B, Papeix C, Tourbah A, Lubetzki C, Lehericy S, Stankoff B (2014). Brain networks disconnection in early multiple sclerosis cognitive deficits: an anatomofunctional study. Hum Brain Mapp.

[17] Mesaros S, Rocca MA, Kacar K, Kostic J, Copetti M, Stosic-Opincal T, Preziosa P, Sala S, Riccitelli G, Horsfield MA, Drulovic J, Comi G, Filippi M (2012). Diffusion tensor MRI tractography and cognitive impairment in multiple sclerosis. Neurology.

[18] Muthuraman M, Fleischer V, Kolber P, Luessi F, Zipp F, Groppa S (2016). Structural brain network characteristics can differentiate CIS from early RRMS. Front Neurosci.

[19] Popescu V, Klaver R, Voorn P, Galis-de Graaf Y, Knol DL, Twisk JW, Versteeg A, Schenk GJ, Van der Valk P, Barkhof F, De Vries HE, Vrenken H, Geurts JJ (2015). What drives MRI-measured cortical atrophy in multiple sclerosis?. Mult Scler.

[20] Ravasz E, Barabasi AL (2003). Hierarchical organization in complex networks. Phys Rev E Stat Nonlinear Soft Matter Phys.

[21] Ravasz E, Somera AL, Mongru DA, Oltvai ZN, Barabasi AL (2002). Hierarchical organization of modularity in metabolic networks. Science.

[22] Rubinov M, Sporns O (2010). Complex network measures of brain connectivity: uses and interpretations. Neuroimage.

[23] Rubinov M, Ypma RJ, Watson C, Bullmore ET (2015). Wiring cost and topological participation of the mouse brain connectome. Proc Natl Acad Sci USA.

[24] Scholtens LH, Schmidt R, de Reus MA, van den Heuvel MP (2014). Linking macroscale graph analytical organization to microscale neuroarchitectonics in the macaque connectome. J Neurosci.

[25] Seewann A, Kooi EJ, Roosendaal SD, Barkhof F, van der Valk P, Geurts JJ (2009). Translating pathology in multiple sclerosis: the combination of postmortem imaging, histopathology and clinical findings. Acta Neurol Scand.

[26] Shu N, Duan Y, Xia M, Schoonheim MM, Huang J, Ren Z, Sun Z, Ye J, Dong H, Shi FD, Barkhof F, Li K, Liu Y (2016). Disrupted topological organization of structural and functional brain connectomes in clinically isolated syndrome and multiple sclerosis. Sci Rep.

[27] Shu N, Liu Y, Li K, Duan Y, Wang J, Yu C, Dong H, Ye J, He Y (2011). Diffusion tensor tractography reveals disrupted topological efficiency in white matter structural networks in multiple sclerosis. Cereb Cortex.

[28] Sporns O (2012). From simple graphs to the connectome: networks in neuroimaging. Neuroimage.

[29] Stam CJ (2014). Modern network science of neurological disorders. Nat Rev Neurosci.

[30] Steenwijk MD, Daams M, Pouwels PJ, L JB, Tewarie PK, Geurts JJ, Barkhof F, Vrenken H (2015). Unraveling the relationship between regional gray matter atrophy and pathology in connected white matter tracts in long-standing multiple sclerosis. Hum Brain Mapp.

[31] Stettler DD, Das A, Bennett J, Gilbert CD (2002). Lateral connectivity and contextual interactions in macaque primary visual cortex. Neuron.

[32] Tononi G, Sporns O, Edelman GM (1994). A measure for brain complexity: relating functional segregation and integration in the nervous system. Proc Natl Acad Sci U S A.

[33] Tzourio-Mazoyer N, Landeau B, Papathanassiou D, Crivello F, Etard O, Delcroix N, Mazoyer B, Joliot M (2002). Automated anatomical labeling of activations in SPM using a macroscopic anatomical parcellation of the MNI MRI single-subject brain. Neuroimage.

[34] van den Heuvel MP, Scholtens LH, de Reus MA, Kahn RS (2016). Associated microscale spine density and macroscale connectivity disruptions in schizophrenia. Biol Psychiatry.

[35] van den Heuvel MP, Scholtens LH, Feldman Barrett L, Hilgetag CC, de Reus MA (2015). Bridging cytoarchitectonics and connectomics in human cerebral cortex. J Neurosci.

[36] Yuste R, Tank DW (1996). Dendritic integration in mammalian neurons, a century after Cajal. Neuron.

