# Supplementary material for: Structural network topology relates to tissue properties in multiple sclerosis
Source: J Neurol. 2018 Nov 22;266(1):212–22. doi: 10.1007/s00415-018-9130-2 (PMC6342882; doi:10.1007/s00415-018-9130-2)
Supplement: Supplementary file 1 — Supplementary material 1 (DOCX 72 KB) [file 415_2018_9130_MOESM1_ESM.docx]

**E-METHODS**

**Subjects**

Eight subjects with MS were included (Table 1). Of each subject, histological data and *in situ post-mortem* (PM) MRI data were collected with a very short PM delay (median=4 [range=2.5–5 hours]). This data was collected in collaboration with the Netherlands Brain Bank. The study was approved by the institutional ethics review board. Before death, the subjects or their next of kin provided written informed consent for the use of their tissue and clinical information for research purposes to the Netherlands Brain Bank. A detailed description of the tissue collection pipeline and *post-mortem* imaging, as well as detailed information about the staining process, microscopy and image analysis, can be found elsewhere but will be summarized below ^1^. Furthermore, an atlas of WM connections was constructed based on an *in vivo* imaging dataset of eight age- and sex-matched healthy controls (5 males, 3 females, median age=61.5 [range=59-63]). Written informed consent was obtained from all healthy controls to participate in this study.

***Post-mortem* MRI acquisition**

For each subject, post-mortem in situ whole-brain MRI was acquired using a 1.5T whole-body scanner (either Siemens Sonata (N=5) or Siemens Avanto (N=3), both Erlangen Germany, (depending on scanner availability at the time of autopsy) with an 8-channel head coil. The protocol included a dual-echo T2-weighted sequence to determine WM lesion volumes (repetition time 5640ms, echo time 22/98ms, slice thickness 3.0mm, in-plane resolution 1.0x1 .0 mm^2^). A 3DT1-weighted fast spoiled gradient echo (FSPGR) sequence (repetition time 2700ms, echo time 5.0ms, inversion time 950ms, slice thickness 1.3mm, in-plane resolution 1.2x1.2 mm^2^) and 2D echo-planar diffusion tensor imaging (DTI) was performed (repetition time 8500ms, echo time 86ms, slice thickness 2.0mm, in-plane resolution 2.0x2.0 mm^2^). The DTI sequence included 60 volumes with non-collinear diffusion gradients (b-value 700 s/mm^2^) and 10 volumes without diffusion weighting.

**Construction of a group-based structural connectivity atlas in healthy controls**

A structural connectivity atlas was constructed in healthy subjects to overcome the potentially confounding effect of MS related WM lesions on tractography methods. In these healthy controls a 3T MRI system (General Electrics, USA) was used to acquire 3DT1 (repetition time 7.8ms, echo time 3.0ms, inversion time 450ms, slice thickness 1.0mm, in-plane resolution 0.9x0.9 mm^2^) and diffusion-weighted (repetition time 13000ms, echo time 91ms, slice thickness 2.4mm, in-plane resolution 2.0x2.0 mm^2^) images. The diffusion-weighted images were corrected for motion and eddy current distortion using FMRIB’s Diffusion Toolbox (FSL-FDT; part of FSL 5.0.9 https://fsl.fmrib.ox.ac.uk/fsl/fslwiki ^2^). Cortical GM regions (*i.e.* nodes) were segmented using the automated anatomical labeling (AAL) atlas ^3^ and FIRST (part of FSL) was used to delineate deep GM, constituting in total 92 nodes. First, bedpostx was run to build up diffusion parameter distributions at each voxel, after which probabilistic tractography was conducted (probtrackx2, part of FSL, 5000 streamlines per voxel) to obtain probabilistic maps of WM connections running between all pairs of nodes resulting in a structural network for each subject (Fig. 1A). The probabilistic maps were then binarized at 0.25% of the total number of generated streamlines passing through both the seed and target mask to ensure consistency across subjects ^4^. After binarization, all subject-specific WM connections in DTI space were non-linearly registered to T1 standard Montreal Neurological Institute (MNI) template space in order to construct a probabilistic atlas of WM connections (92x91) by summing all individuals’ masks into a common mask. To further reduce the number of false positives, only voxels in which a tract was present in at least six out of eight healthy controls were included in the final binarized atlas ^5^ (Fig. 1A).

**Construction of individual structural connectomes in MS subjects**

The *post-mortem* *in situ* diffusion images were corrected for motion and eddy current distortion using FSL-FDT. Then the diffusion tensor was fitted and fractional anisotropy (FA) was computed for each voxel. In order to construct structural networks in subjects with MS, the previously obtained average healthy control atlas of WM connections was then applied to the FA maps of the MS subjects. To optimize registration pipelines, all WM lesions were manually outlined on the T2 images using MIPAV (http://mipav.cit.nih.gov/). T2 lesion masks were linearly registered to 3DT1 images using FLIRT, subsequently lesion-filling was performed using LEAP ^6^. After that, non-linear registration parameters from 3DT1 subject space to MNI space were calculated using FNIRT (part of FSL5.0.9). The obtained warp was inverted in order to non-linearly co-register the atlas of WM connections to the lesion filled 3DT1-weighted images ^4 7 8^. These co-registrations were visually assessed for accuracy. To ensure the inclusion of WM only, the mask of each WM connection was separately multiplied with each subjects’ lesion-filled WM mask derived from SIENAX. Subsequently, the resulting masks of the WM connections were linearly registered to the diffusion-weighted images. The quality of this registration was also visually inspected. Mean FA values were then extracted for all WM connections, constituting a FA matrix per subject. To obtain a binary structural connectivity matrix per subject, individual FA matrices were binarized at a density of 20% ^9^, by only including WM connections with the top 20% highest mean FA values (Fig. 1B).

**Computation of topological macro-scale measures**

Using the structural connectivity matrix of each subject, macro-scale network topology was computed for each of the 92 regions using the Brain Connectivity Toolbox ^10^ and in-house developed Matlab scripts (Matlab version 13a, Mathworks, Natick, MA, USA). Regional macro-scale topological properties of segregation and integration were examined. Several graph theoretical measures of segregation exist, but we chose to adhere to the classical measure of clustering coefficient ^11^. It quantifies the extent of connectivity occurring in triangles, *i.e.* to which extent the neighbors of a particular region are also connected to each other (Fig. 1B). Classically path length is the graph theoretical measures of integration ^12^. However, connection distance between brain regions has also been implicated in integrative processes, *i.e.* (WM) connections between regions that are spatially far apart ^13-16^. For integration, we used average fiber length as a structural indicator of integrative tendency per region. The fiber length was determined per connection in the healthy controls by additional weighting for fiber length during tractography. This fiber length matrix was then masked by the connectivity matrix of each patient to determine the average fiber length of WM fibers per node for every patient (Fig. 1B). This means that the difference in fiber length between patients is based on which connections were part of their network based on FA value of the tracts. Whole-brain measures of the clustering coefficient and fiber length were calculated by averaging the values of all nodes for each patient.

**Tissue selection and cellular micro-scale measures**

To quantify micro-scale regional features, five cortical brain regions were excised according to a standardized protocol ^17^. The superior frontal gyrus (N=8), inferior frontal gyrus (N=6), cingulate gyrus (N=7), inferior parietal lobule (N=7) and superior temporal gyrus (N=7) were excised, adding up to a total of 33 tissue blocks across all subjects (Fig. 1C). From now on these will be referred to as regions of interest (ROIs). Twenty-nine tissue blocks were taken from the left hemisphere and four tissue blocks were taken from the right hemisphere. Tissue blocks were formalin fixed and embedded in paraffin. Subsequently, tissue sections were cut at 10µm thickness prior to staining. Immunohistochemistry was performed to stain for four different micro-scale cell properties: 1) neuronal density (*i.e.* neuronal count per mm^2^), 2) neuronal size in µm^2^ (*i.e.* total area stained for neurons divided by the neuronal number, per mm^2^), 3) axonal density in relative optical density (ROD) and 4) total cell density (*i.e.* the sum of the number of astrocytes, neurons and oligodendrocytes in number per mm^2^; Fig. 1D). Myelin density was obtained to identify whether it was related to the above mentioned micro-scale measures.

**Staining procedure**

Neurons were stained using an anti-NeuN antibody (1:1000; Chemicon, MAB377), axons were stained using an anti-SMI312 antibody (1:2000; Covance, SMI-311R), oligodendrocytes were stained using an anti-olig2 antibody (1:500; Chemicon, AB9610), astrocytes were stained using an anti-GFAP antibody (1:500; Sigma, G3893) and myelin was stained using an anti-PLP antibody (1:500; Serotec, MCA839G). Antigen retrieval was performed prior to staining using a citrate buffer (pH6) for all stainings, except for the olig2 staining where Tris-EDTA (pH9) was used as a pre-treatment. Sections were blocked with normal goat serum and after incubation with the primary antibodies sections were rinsed and incubated with biotin labeled secondary antibodies (1:500 DAKO, Glostrup, Denmark) then they were rinsed again and incubated with streptavidin-biotin-peroxidase complexes (1:200; Vectastain; Vector Labotaries Inc., Burlingame, CA, USA). Only during the olig2 staining Envision horseradish peroxidase complexes (DAKO, Glostrup, Denmark) was used instead of a regular biotinylated secondary antibodies and streptavidin-biotin-peroxidase complexes. Finally, sections were rinsed and 3,3’-diaminobenzidine tetrahydrochloride dehydrate (DAB; DAKO, Glostrup, Denmark) precipitate was generated in reaction with peroxidase.

**Acquisition and quantification of cellular micro-scale measures**

Images of were acquired using a Leica DM/RBE photomicroscope (Leica, Heidelberg, Germany). A 4 × 4 mm^2^ grid was overlayed on an entirely imaged Nissl stained section and by means of random systematic sampling grid frames containing six layered cortex were selected as quantification sites for every tissue block. These sites were the same for all micro-scale measures quantified in consecutive sections per tissue block. The number sites analyzed varied according to the size of the section and per micro-scale measure an average was taken of all sites analyzed in a section. Neuronal density, size and astrocyte density were quantified using MCID segmentation scripts (MCID Image Analysis Software Solutions for Life Sciences, UK), while the number of oligodendrocytes was quantified using ImageJ software. Axonal density was quantified using the relative optical density (ROD), which is a method that measures the amount of stained structures based on the optical staining density using conversion of the image to a grayscale image and correcting for the background intensity ^1^. Finally, the extent of cortical (de)myelination was quantified using the ROD.

**Statistical analysis**

Statistical analyses were performed using Matlab and SPSS (version 22.0, IBM, Chicago, IL, USA). Spearman's rank correlation coefficient was used to evaluate the correlations between (a) macro-scale network properties, (b) regional macro-scale network properties with their corresponding micro-scale histological features and (c) whole brain WM lesion volume and regional GM demyelination with macroscopic and microscopic measures. To evaluate the possible effect of age, we performed a partial correlation including age as a covariate. *P*-values were considered significant at *p*<0.050 and two-tailed testing was performed. In addition to these non-parametric statistics, we performed permutation testing to further objectify the significance of the correlations between micro-scale and macro-scale measures. In permutation testing, all micro-scale and macro-scale measures were randomly shuffled 1000 times, forming a distribution of *p*-values and coefficients based on these correlations. We then ascertained that the *p*-value of our actual statistical test fell within the upper 5% of this distribution. Furthermore, with respect to the connection density threshold for each individual connectome (20% in our main analyses), reproducibility of all results was tested across different density thresholds (namely 20%, 25% and 30%). Further, regional specificity of correlations between the micro-scale and macro-scale regional properties was examined by random resampling. To do so, we resampled our data within subjects using macro-scale network topological measures of all 92 regions and randomly coupled these to the micro-scale characteristics for each of the five investigated cortical regions (N=33 across subjects). In doing so, the micro-scale features of a certain region (*e.g.* superior frontal gyrus) were not necessarily paired with macro-scale indices of the same region, but randomly assigned to macro-scale characteristics of one of the other 91 regions. This resampling was repeated 1000 times across subjects and averaged to obtain a *p*-value and effect size for the resampled data. This analysis quantified whether the observed correlations were region specific or were a global property of the network and therefore true for all regions constituting the structural network (*i.e.* all 92 GM regions). This analysis was only performed for the correlation between neuronal size and clustering coefficient.

**REFERENCES**

1. Popescu V, Klaver R, Voorn P, et al. What drives MRI-measured cortical atrophy in multiple sclerosis? *Mult Scler* 2015;21(10):1280-90. doi: 10.1177/1352458514562440

2. Jenkinson M, Beckmann CF, Behrens TE, et al. Fsl. *Neuroimage* 2012;62(2):782-90. doi: 10.1016/j.neuroimage.2011.09.015

3. Tzourio-Mazoyer N, Landeau B, Papathanassiou D, et al. Automated anatomical labeling of activations in SPM using a macroscopic anatomical parcellation of the MNI MRI single-subject brain. *Neuroimage* 2002;15(1):273-89. doi: 10.1006/nimg.2001.0978

4. Daams M, Steenwijk MD, Wattjes MP, et al. Unraveling the neuroimaging predictors for motor dysfunction in long-standing multiple sclerosis. *Neurology* 2015;85(3):248-55. doi: 10.1212/WNL.0000000000001756

5. Kolasinski J, Stagg CJ, Chance SA, et al. A combined post-mortem magnetic resonance imaging and quantitative histological study of multiple sclerosis pathology. *Brain* 2012;135(Pt 10):2938-51. doi: 10.1093/brain/aws242

6. Chard DT, Jackson JS, Miller DH, et al. Reducing the impact of white matter lesions on automated measures of brain gray and white matter volumes. *J Magn Reson Imaging* 2010;32(1):223-8. doi: 10.1002/jmri.22214

7. Daams M, Steenwijk MD, Schoonheim MM, et al. Multi-parametric structural magnetic resonance imaging in relation to cognitive dysfunction in long-standing multiple sclerosis. *Mult Scler* 2016;22(5):608-19. doi: 10.1177/1352458515596598

8. Steenwijk MD, Daams M, Pouwels PJ, et al. Unraveling the relationship between regional gray matter atrophy and pathology in connected white matter tracts in long-standing multiple sclerosis. *Hum Brain Mapp* 2015;36(5):1796-807. doi: 10.1002/hbm.22738

9. Eavani H, Satterthwaite TD, Filipovych R, et al. Identifying Sparse Connectivity Patterns in the brain using resting-state fMRI. *Neuroimage* 2015;105:286-99. doi: 10.1016/j.neuroimage.2014.09.058

10. Rubinov M, Sporns O. Complex network measures of brain connectivity: uses and interpretations. *Neuroimage* 2010;52(3):1059-69. doi: 10.1016/j.neuroimage.2009.10.003

11. Watts DJ, Strogatz SH. Collective dynamics of 'small-world' networks. *Nature* 1998;393(6684):440-2. doi: 10.1038/30918

12. Sporns O. Structure and function of complex brain networks. *Dialogues Clin Neurosci* 2013;15(3):247-62.

13. Alexander-Bloch A, Lambiotte R, Roberts B, et al. The discovery of population differences in network community structure: new methods and applications to brain functional networks in schizophrenia. *Neuroimage* 2012;59(4):3889-900. doi: 10.1016/j.neuroimage.2011.11.035

14. Bassett DS, Greenfield DL, Meyer-Lindenberg A, et al. Efficient physical embedding of topologically complex information processing networks in brains and computer circuits. *PLoS Comput Biol* 2010;6(4):e1000748. doi: 10.1371/journal.pcbi.1000748

15. Markov NT, Ercsey-Ravasz M, Lamy C, et al. The role of long-range connections on the specificity of the macaque interareal cortical network. *Proc Natl Acad Sci U S A* 2013;110(13):5187-92. doi: 10.1073/pnas.1218972110

16. Meunier D, Lambiotte R, Bullmore ET. Modular and hierarchically modular organization of brain networks. *Front Neurosci* 2010;4:200. doi: 10.3389/fnins.2010.00200

17. Seewann A, Kooi EJ, Roosendaal SD, et al. Translating pathology in multiple sclerosis: the combination of postmortem imaging, histopathology and clinical findings. *Acta Neurol Scand* 2009;119(6):349-55. doi: 10.1111/j.1600-0404.2008.01137.x
